# Diagnosis of post-neurosurgical bacterial meningitis in patients with aneurysmal subarachnoid hemorrhage based on the immunity-related proteomics signature of the cerebrospinal fluid

**DOI:** 10.3389/fneur.2023.1166598

**Published:** 2023-06-20

**Authors:** Liang Zhao, Pingping Li, Ziao Xu, Xuefei Ji, Liao Guan, Xiaojian Wang, Jin Luo, Hongwei Cheng, Lei Ye

**Affiliations:** Department of Neurosurgery, The First Affiliated Hospital of Anhui Medical University, Hefei, China

**Keywords:** post-neurosurgical bacterial meningitis, proteomics, aneurysmal subarachnoid hemorrhage, immunity, neuroinflammation

## Abstract

**Introduction:**

Post-neurosurgical bacterial meningitis (PNBM) is a serious complication for patients who receive neurosurgical treatment, but the diagnosis is difficult given the complicated microenvironment orchestrated by sterile brain injury and pathogenic infection. In this study, we explored potential diagnostic biomarkers and immunological features using a proteomics platform.

**Methods:**

A total of 31 patients with aneurysmal subarachnoid hemorrhage (aSAH) who received neurosurgical treatment were recruited for this study. Among them, 15 were diagnosed with PNBM. The remaining 16 patients were categorized into the non-PNBM group. Proteomics analysis of the cerebrospinal fluid (CSF) was conducted on the Olink platform, which contained 92 immunity-related molecules.

**Results:**

We found that the expressions of 27 CSF proteins were significantly different between the PNBM and non-PNBM groups. Of those 27 proteins, 15 proteins were upregulated and 12 were downregulated in the CSF of the PNBM group. The receiver operating characteristic curve analysis indicated that three proteins (pleiotrophin, CD27, and angiopoietin 1) had high diagnostic accuracy for PNBM. Furthermore, we also performed bioinformatics analysis to explore potential pathways and the subcellular localization of the proteins.

**Conclusion:**

In summary, we found a cohort of immunity-related molecules that can serve as potential diagnostic biomarkers for PNBM in patients with aSAH. These molecules also provide an immunological profile of PNBM.

## Introduction

Post-neurosurgical bacterial meningitis (PNBM) is a common complication in patients undergoing neurosurgical treatments (1). Despite the application of aseptic technology worldwide, the occurrence of PNBM appears inevitable, with the incidence rate ranging between 0.3 and 10% in different neurosurgical diseases (2, 3). Aneurysmal subarachnoid hemorrhage (aSAH), although it only accounts for 10%−20% of all strokes, is a serious cerebrovascular disorder that may cause considerable morbidity and mortality (4). The prognosis of aSAH patients depends on multiple factors, such as the severity of the acute bleed and the presence of delayed cerebral ischemia and post-neurosurgical complications. Among these, PNBM is one of the most common complications encountered in clinical practice. Despite the lack of epidemiological information regarding the incidence of PNBM among aSAH patients, a report indicated that the general PNBM incidence in cerebrovascular diseases is ~4.9% (42/852) (3). The gold standard diagnostic criteria for PNBM depend on Gram's staining of the cerebrospinal fluid (CSF) or bacterial culture. However, low bacterial loads in the CSF and the pre-application of antibiotics usually lead to negative results in clinical practice. Previous studies have shown that the positive rates for CSF bacterial culture are <20% in different laboratory tests (5, 6). In reality, the biochemical characteristics of CSF predominantly assist in the clinical diagnosis of bacterial infection of the central nervous system (CNS). In comparison with community-acquired CNS infection, the microenvironment of the CSF in PNBM is more complicated because primary neurological disease, neurosurgical processes, and pathogenic infection have a significant impact on numerous aspects of the biological functions of the CSF, such as immune reaction and inflammation. Investigation of biomolecule-based diagnostic biomarkers of PNBM has, therefore, always been a focus in clinical studies and scientific research.

The CNS is considered one of the immune-privileged areas with respect to the peripheral immunological system because of multiple barriers, such as the blood–brain barrier (BBB) and the meninges around the neural parenchyma (7). However, the involvement of both the innate and adaptive immune systems, which maintain CNS homeostasis, has been reported in both CNS infection and sterile brain injury (8). Numerous immunocytes, such as localized macrophages, peripherally recruited leukocytes, and CD4^+^/CD8^+^ T cells, can participate in the neuroinflammatory reaction, pathogen cleanup, and damage repair. Cytokines and chemokines that are released by immunocytes serve as diagnostic and/or predictive biomarkers for infectious disease and sterile brain injuries (9).

Neuroinflammation is one of the featured pathogenesis of secondary brain injury in stroke (10). Molecules that function as both proinflammatory and anti-inflammatory mediators are extensively produced and released by CNS-resident cells and immunocytes that are recruited from the peripheral circulation. However, some studies have shown that CNS infection can also induce extensive neuroinflammation (11, 12). However, the immunological reactions associated with the comorbidity of CNS infection and hemorrhagic stroke have not been fully investigated. Therefore, although CNS infections share a similar pathogenetic process as neuroinflammatory reactions, it is still largely unknown whether these inflammation-related molecules can serve as potential diagnostic biomarkers in PNBM among patients with stroke.

In this study, we used a novel proteomics platform that contains 92 immunity-related biomarkers to detect CNS biomarkers for the diagnosis of PNBM. We believe that this proteomics analysis can provide not only a molecule-based diagnosis of PNBM but also an immunological profile for the comorbidity of PNBM and aSAH.

## Methods

### Patients and sample collection

A total of 31 patients with aSAH were recruited for this study. The diagnosis of aSAH was confirmed by two senior neurosurgeons with supporting evidence from computed tomography and whole cerebrovascular digital subtraction angiography. All patients underwent computed tomography angiography (CTA) after admission to assess the incidence of intracranial aneurysms (IA). Cistern drainage operations were performed for all patients to release the bloody CSF. A total of 10 and 21 patients underwent clipping and endovascular embolization of IAs, respectively. When cistern drainage was completed, some patients exhibited potential infection symptoms. Therefore, 20 patients received another lumbar puncture for laboratory testing of CSF. PNBM was subsequently diagnosed in 15 out of 31 patients based on the diagnostic criteria issued by IDSA's Clinical Practice Guidelines for Healthcare-Associated Ventriculitis and Meningitis 2017 (13) and a Chinese Expert Consensus of Diagnostic and Therapy for the Neurosurgical Central Nervous System Infections in 2021. According to the guidelines, PNBM diagnosis briefly relies on either positive results of Gram's staining/bacterial culture or the CSF indications (simultaneously satisfying CSF white blood cells > 100 × 10^6^/L. CSF glucose <2.2 mmol/L, and CSF-to-blood glucose ratio <0.4). The inclusion criteria were as follows: (1) aSAH patients that received neurosurgical treatments (such as endovascular embolization or clipping of IAs); (2) patients with complete demographic and clinicopathological data; and (3) an adequate amount and quality of CSF samples available for proteomics analysis. The exclusion criteria were as follows: (1) patients with other types of hemorrhagic stroke (e.g., spontaneous intracerebral hemorrhage, arteriovenous malformation, and traumatic-induced intracranial hemorrhage); (2) inadequate amount or quality of CSF samples available for the study (e.g., severely hemolyzed or contaminated sample during transportation or storage; (3) patients who had other comorbid neurological diseases; and (4) patients who had systemic inflammatory diseases or malignant tumors. CSF samples were extracted via cistern drainage or lumbar puncture with an aseptic technique when the patients were suspected of having potential CNS infections. All samples were stored at −80°C. The demographic data and biochemical characteristics of CSF are summarized in Table 1.

**Table 1:** Demographic and clinicopathological data of the 31 patients with aneurysmal subarachnoid hemorrhage.

	**Total (*n* = 31)**	**Infection (*n* = 15)**	**Non-infection (*n* = 16)**	***p*-value**
Gender (M/F, *n*)	31	9/6	5/11	0.108
Age	55.74 ± 15.50	57.47 ± 14.41	54.13 ± 16.77	0.558
ICP (mmH_2_O)	165.16 ± 50.06	192.00 ± 50.17	140.00 ± 35.59	0.002
CSF WBC	322 [105, 901]	572 [322, 1,602]	151 [13, 690]	0.005
CSF total protein	2.11 [1.25, 3.00]	2.86 [2.02, 3.00]	1.48 [0.57, 2.39]	0.002
CSF RBC	28,000 [2,000, 101,000]	24,000 [1,000, 200,000]	40,500 [2,000, 95,500]	0.921
CSF glucose	2.77 ± 1.53	1.53 ± 0.40	3.94 ± 1.24	<0.001
CSF chlorine	121.42 ± 10.50	120.61 ± 10.85	122.17 ± 10.46	0.688
CSF polymorphonuclear cells	52.82 ± 28.28	66.49 ± 24.85	40.00 ± 25.71	0.007
Blood glucose	6.19 [5.51, 7.77]	6.19 [5.57, 7.68]	6.26 [5.17, 7.96]	0.968
CSF-to-blood glucose ratio	0.35 [0.22, 0.57]	0.23 [0.18, 0.33]	0.56 [0.44, 0.64]	<0.001
Hunt-Hess Score	2 [1, 4]	2 [1, 3]	3 [2, 4]	0.037
Glasgow Coma Scale	15 [12, 15]	15 [15, 15]	15 [12, 15]	0.086
Location	Anterior communicating artery	3	5	0.058
	Middle cerebral artery	6	5	
	Posterior communicating artery	1	6	
	Internal carotid artery	4	0	
	Anterior cerebral artery	1	0	

### Proximity extension assay

An immunity-related panel containing 92 molecules was measured in the CSF of all participants using Proximity Extension Assay (PEA) technology on the Olink^®^ Proteomics Multiplex Assay platform (14). The assays were performed using Sinotech Genomics Co. Ltd. (Shanghai, China). Briefly, antibodies that specifically recognized target proteins were designed by conjugating DNA tags at the end. DNA tags hybridize to form paired double strands when the antibodies correctly match the protein. The DNA tag sequences were amplified using qPCR for quantitative detection. The results were expressed as normalization protein expression (NPX) values on a log_2_-scale. The molecules contained in the platform were adenosine deaminase (ADA), adhesion G protein-coupled receptor G1 (ADGRG1), angiopoietin 1 (ANGPT1), angiopoietin 2 (ANGPT2), arginase 1 (ARG1), carbonic anhydrase IX (CAIX), caspase 8 (CASP-8), C-C motif chemokine 3 (CCL3), CCL4, CCL17, CCL19, CCL20, CCL23, CD4, CD5, CD8A, CD27, CD28, CD40, CD40 ligand (CD40L), CD70, CD83, CD244, cytotoxic and regulatory T-cell molecule (CRTAM), macrophage colony-stimulating factor 1 (CSF-1), fractalkine (CX3CL1), C-X-C motif chemokines (CXCL1), CXCL5, CXCL9, CXCL10, CXCL11, CXCL12, CXCL13, decorin (DCN), pro-epidermal growth factor (EGF), tumor necrosis factor ligand superfamily member 6 (FASLG), fibroblast growth factor 2 (FGF2), galectin-1 (Gal-1). Gal-9, granzyme A (GZMA), granzyme B (GZMB), granzyme H (GZMH), hepatocyte growth factor (HGF), heme oxygenase 1 (HO-1), ICOS ligand (ICOSLG), interferon γ (IFN-γ), interleukin 1α (IL-1α), IL-2, IL-4, IL-5, IL-6, IL-7, IL-8, IL-10, IL-12, interleukin-12 receptor subunit β-1 (IL-12RB1), IL-13, IL-15, IL-18, IL-33, killer cell immunoglobulin-like receptor 3DL1 (KIR3DL1), natural killer cells antigen CD94 (KLRD1), Lymphocyte activation gene 3 protein (LAG3), lysosome-associated membrane glycoprotein 3 (LAMP3), transforming growth factor beta-1 proprotein (LAPTGF-β1), C-C motif chemokine 2 (MCP-1), C-C motif chemokine 8 (MCP-2), C-C motif chemokine 7 (MCP-3), C-C motif chemokine 13 (MCP-4), MHC class I polypeptide-related sequence A/B (MIC-A/B), matrilysin (MMP-7), macrophage metalloelastase (MMP-12), mucin-16 (MUC-16), natural cytotoxicity triggering receptor 1 (NCR1), nitric oxide synthase 3 (NOS3), programmed cell death protein 1 (PDCD1), platelet-derived growth factor subunit B (PDGF subunit B), programmed cell death 1 ligand 1 (PD-L1), PD-L2, placenta growth factor (PGF), pleiotrophin (PTN), angiopoietin-1 receptor (TIE2), tumor necrosis factor (TNF), tumor necrosis factor receptor superfamily member 4 (TNFRSF 4), TNFRSF 9, TNFRSF12A, TNFRSF21, TNFSF14, tumor necrosis factor ligand superfamily member 10 (TRAIL), tumor necrosis factor ligand superfamily member 12 (TWEAK), vascular endothelial growth factor A (VEGFA), and vascular endothelial growth factor receptor 2 (VEGFR-2).

### Statistical analysis

All statistical analyses were performed in R script (v. 4.0.3), with the primary analysis focusing on the CSF results. All continuous data were depicted as the mean±standard deviation (mean±SD) and then analyzed with the Student's *t*-test or as medians with the interquartile range (IQR) and then analyzed using the Mann–Whitney *U*-test. Binary data were analyzed using the chi-squared test. In the correlation analysis among the molecules in CSF, Pearson's correlation coefficients were assessed for each molecule. A receiver operating characteristic (ROC) curve analysis was conducted to assess the diagnostic efficacy of molecules in PNBM via the index of the area under the curve (AUC). A *p*-value of <0.05 was considered to indicate statistical significance.

## Results

### Quality control of samples

**Figure 1:**
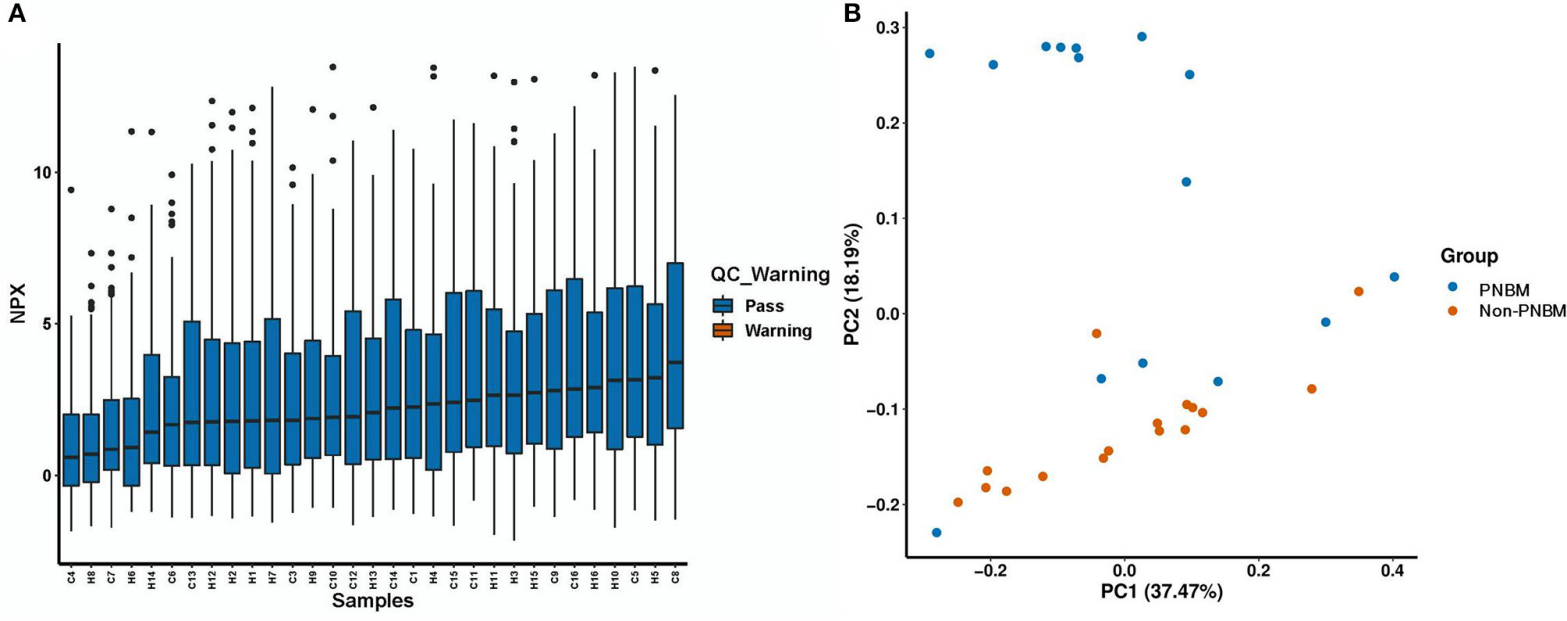
Quality control for 31 samples in the proteomic analysis. **(A)** Normalization Protein eXpression (NPX) analysis. **(B)** Principal component analysis (PCA).

We evaluated 92 protein biomarkers using the Olink Proteomics Multiplex Assay platform. The expression of proteins was summarized using the NPX index to identify the outlier sample. The NPX result for each sample is presented in Figure 1A, which shows that all 31 samples passed quality control. A principle component analysis (PCA) was conducted to assess the results of quality control. Generally, no outliers in the samples were found for all samples in the PCA analysis (Figure 1B). However, we also found that some samples in the case group fell into the category of health samples. This phenomenon might be attributed to individual differences.

### Protein expression profiles

**Figure 2:**
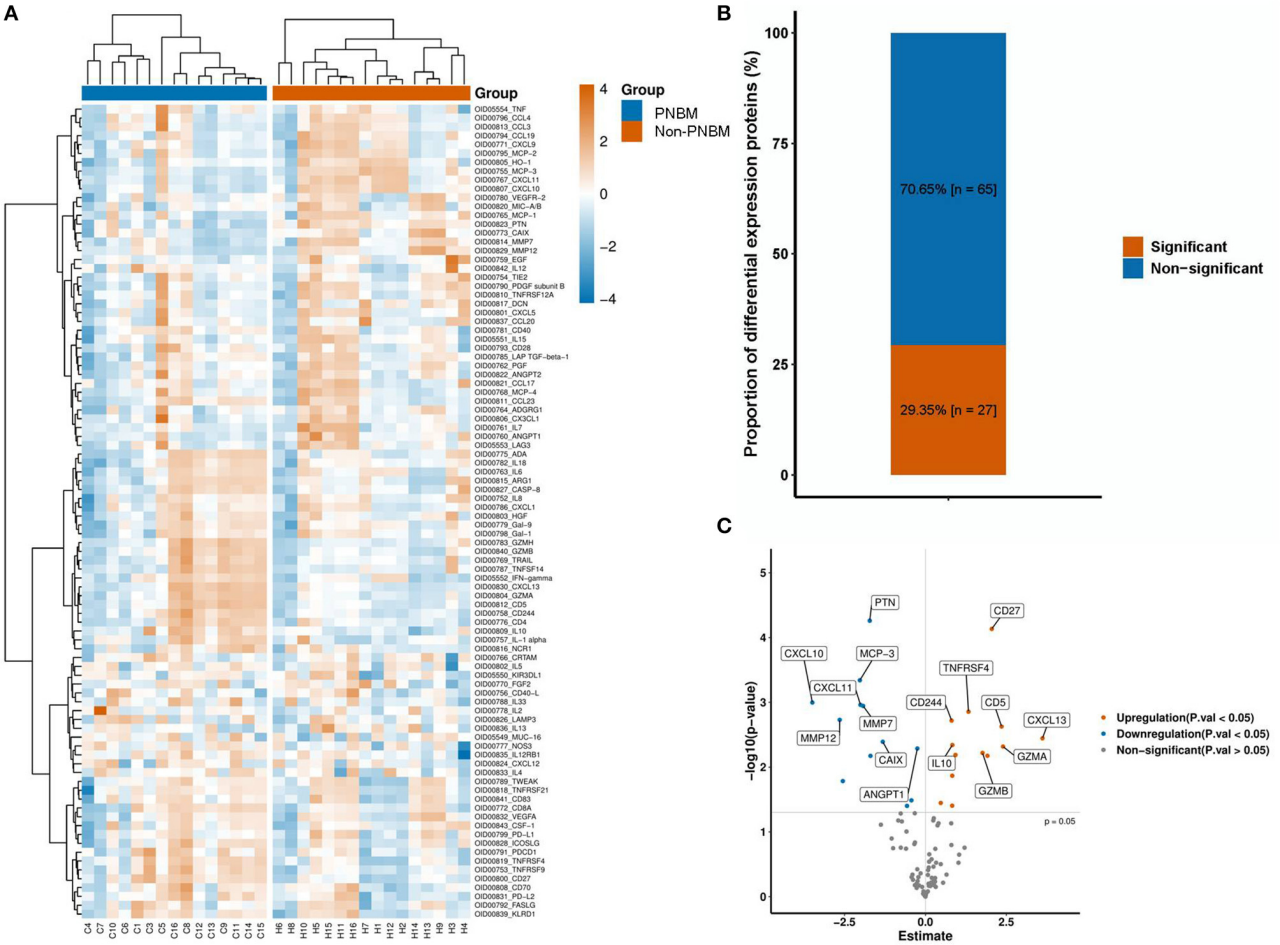
Protein expression profiles between the infection and non-infection groups. **(A)** A heatmap of 92 proteins. **(B)** The proportion of differentially expressed proteins. **(C)** A Volcano plot of the 27 proteins with differential expressions.

All protein expressions in the panel between PNBM patients and non-PNBM patients are depicted in the heatmap (Figure 2A). We found that 27 out of 92 (29.35%) proteins had statistically significant differences between the groups (Figure 2B). Among all the differentially expressed proteins, 15 proteins (CD4, CD5, CD27, CD244, IFN-γ, IL-1α, IL-10, CXCL-13, TNFRSF4, TNFRSF9, TNFSF14, GZMA, GZMB, GZMH, and PDCD1) were upregulated and 12 (CXCL-10, CXCL-11, MMP-7, MMP-12, MCP-1, MCP-3, CAIX, ANGPT1, PTN, HO-1, EGF, and PDGF subunit B) were downregulated in the CSF samples of PNBM patients (Figure 2C) compared to those of non-PNBM patients. We analyzed Pearson's correlation coefficients for all proteins to evaluate the correlations among proteins (Supplementary Figure 1). The results indicated that most proteins were positively correlated with each other.

### Diagnostic efficacy for PNBM biomarkers

**Figure 3:**
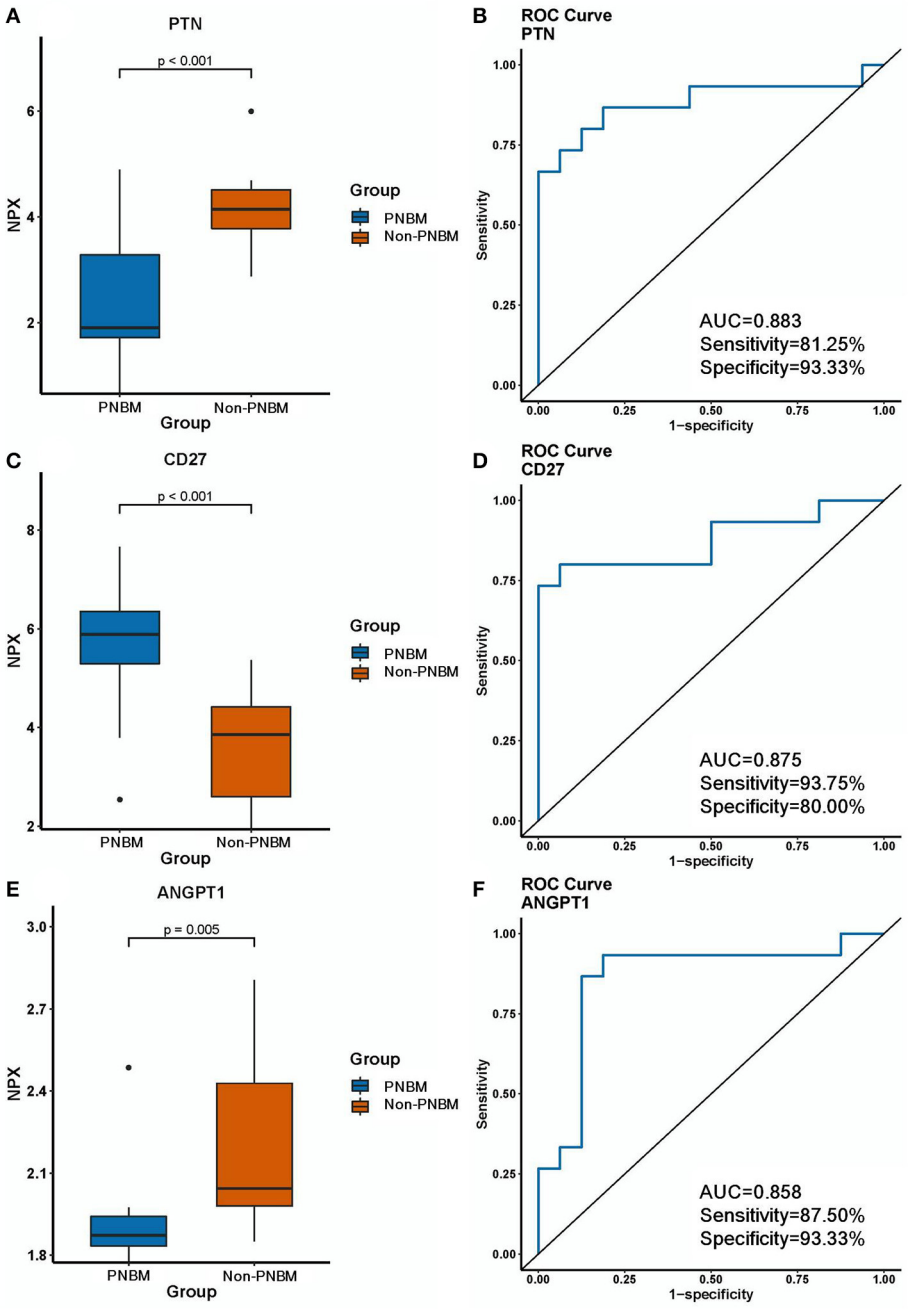
Expression differences and diagnostic efficacy of biomarkers between the infection and non-infection groups. **(A, B)** PTN. **(C, D)** CD27. **(E, F)** ANGPT1.

We analyzed the diagnostic efficacy of the 27 differentially expressed proteins between the PNBM and non-PNBM groups using ROC analysis. The results showed that three proteins reached high diagnostic efficiencies (AUC > 0.85) in distinguishing PNBM from non-PNBM in patients with aSAH; these were PTN (*p* < 0.001, AUC = 0.883, sensitivity = 81.25%, specificity = 93.33%; Figures 3A, B); CD27 (*p* < 0.001, AUC = 0.875, sensitivity = 93.75%, specificity = 80.00%; Figures 3C, D); and ANGPT1 (*p* = 0.005, AUC = 0.858, sensitivity = 87.50%, specificity = 93.33%; Figures 3E, F). Another three proteins (TNFSF14, EGF, and CD4) had mild diagnostic efficacy (AUC: 0.60–0.70), and the other proteins had moderate diagnostic efficacy (AUC: 0.70–0.85) for PNBM.

### Bioinformatic annotations for proteins involving the pathogenesis of PNBM

**Figure 4:**
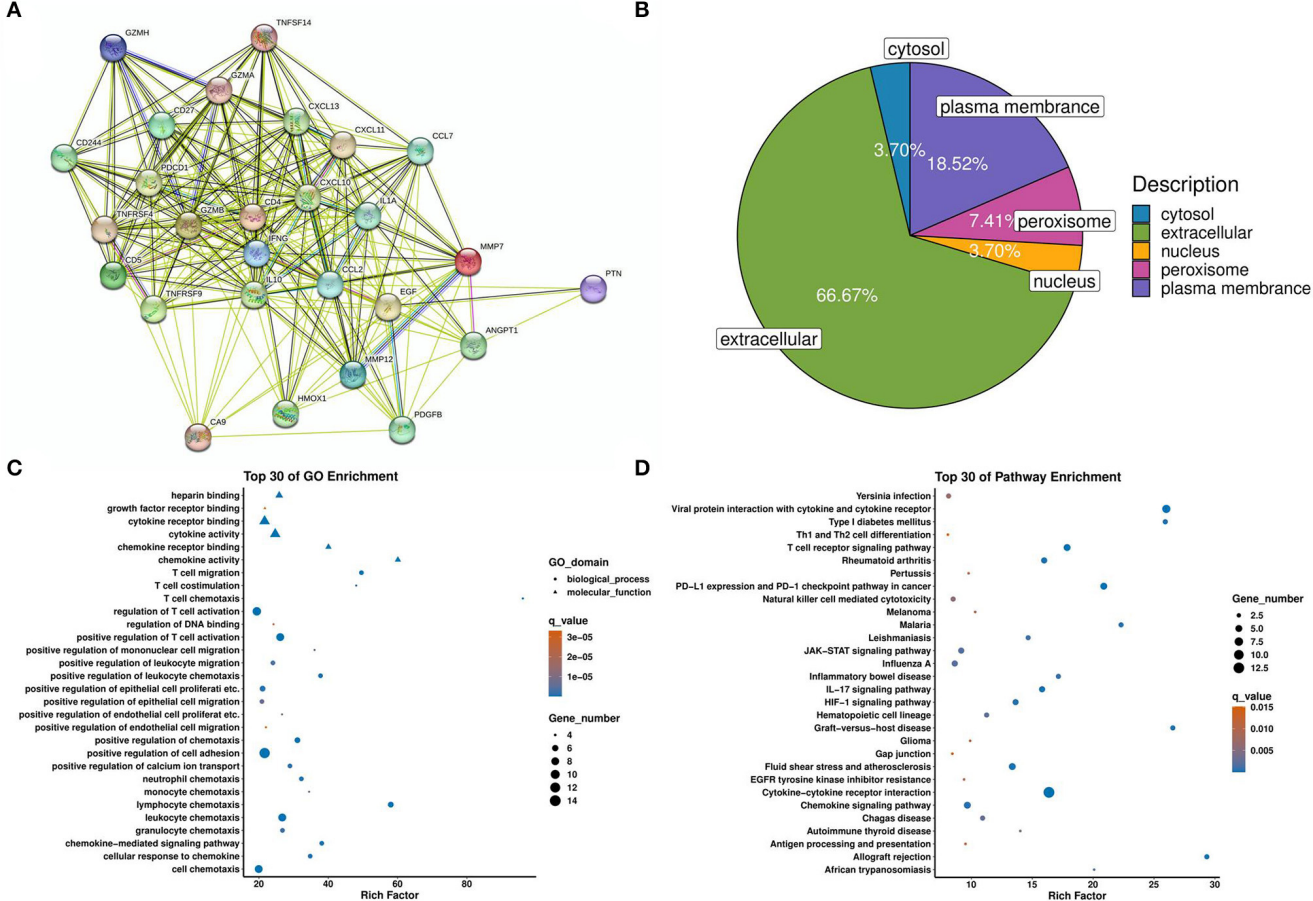
Bioinformatic analysis of differentially expressed proteins. **(A)** Protein-protein interactions. **(B)** Subcellular locations. **(C)** GO enrichment. **(D)** KEGG pathway enrichment.

We used multiple bioinformatics tools to predict the potential protein-protein interaction (PPI), functional pathways, and subcellular locations. First, we established a PPI network to show the interaction among proteins that were differentially expressed between the PNBM and non-PNBM groups (Figure 4A). Then, we analyzed the subcellular locations of these proteins in PNBM (Figure 4B). The results showed that the subcellular locations of the proteins were as follows: extracellular (18 proteins), plasma membrane (five proteins), peroxisome (two proteins), cytosol (one protein), and nucleus (one protein). Additionally, we performed Gene Ontology (GO; Figure 4C) and Kyoto Encyclopedia of Genes and Genomes (KEGG) pathway enrichment (Figure 4D) analyses to show the potential biological functions and pathways involved in the pathogenesis of PNBM among aSAH patients.

## Discussion

In this study, we investigated the immunity-related proteomics profile of the CSF in aSAH patients with or without PNBM using a 92-protein panel. The results showed that 15 upregulated proteins and 12 downregulated proteins were significantly associated with PNBM and could serve as potential biomarkers of the diagnosis.

Owing to the low abundance of microbes in the CSF and the application of antibiotics to patients with potential symptoms of CNS infection, negative results for bacterial culture and Gram staining of the CSF are commonly observed. In our department, the patients who received a clipping of IAs were usually treated with antibiotics intraoperatively and 24-h post-operatively, respectively. This leads to a complicated diagnosis of PNBM among those receiving neurosurgical treatments (15). Recent studies have focused on the discovery of biomarkers for the diagnosis of PNBM. Lactate and procalcitonin (PCT) in the CSF have been found to be suggestive biomarkers for the diagnosis of CNS bacterial infections and have already been clinically applied for the auxiliary diagnosis of meningitis and sepsis (16, 17). Other molecules, such as MMP-9 and a combination of APOE/APOAI/S100A8 proteins, were also shown to play diagnostic roles in community-acquired bacterial meningitis (18, 19). We found that some of these biomarkers were mediators in aSAH or other neurological diseases (20, 21). However, there is a lack of supporting evidence showing their roles in PNBM.

Temporary immunodepression is a common phenomenon among patients with SAH and contributes to the incidence of post-neurosurgical infection. Sarrafzadeh et al. (22) reported considerable immunodepression in the early stages of aSAH and correlated it with a high incidence of pneumonia. Additionally, Chaudhry et al. (23) reported that SAH-induced systemic immunodepression was significantly associated with plasma IL-10, an inflammatory cytokine whose level can be increased after SAH onset. Therefore, exploring the immunity profiles in CSF would help understand immunodepression conditions and provide potential targeted treatment to prevent infectious complications after aSAH.

In our study, we found that, among the 27 proteins that were differentially expressed in CSF between aSAH patients with and without CNS bacterial infections, nine proteins were reported as functional in highly cited studies concerning stroke and primary CNS infection. These proteins included four upregulated molecules (CD4, IFN-γ, IL-1α, and IL-10) and five downregulated molecules (MMP-7, MMP-12, MCP-1, HO-1, and PDGF subunit B). Several previous studies have analyzed CSF proteomics in patients with SAH using different detection platforms (24–26). This proteomics-related biomarker detection helped identify the molecular basis of SAH or its related complications. However, evidence showing the immunity profile of SAH or PNBM following SAH is still lacking. Neuroinflammation is the hallmark of secondary injury after stroke.

Numerous cytokines and chemokines with anti-inflammatory and proinflammatory properties interact with each other and participate in neural damage and repair. The subsequent pathogen-induced neuroinflammatory reaction is also involved in the immunity needed to execute the cleanup of pathogens. Although these two challenges share overlapping immunological features, the inducer differs in origin, leading to different levels of anti-inflammatory and proinflammatory biomarkers (8). In CNS infection, both innate (monocytes and neutrophils) and adaptive immunocytes (CD8^+^, CD4^+^, and B cells) are recruited from peripheral circulation through the damaged BBB. However, innate immunocytes (myelomonocytic cells) are predominantly involved in the damage cleanup and repair initiation when a sterile brain injury occurs (9). Interestingly, we found that the molecules that were reported to be associated with both stroke and CNS bacterial infection had only a mild-to-moderate diagnostic efficacy (AUC range: 0.65–0.84). We inferred that these molecules are influenced by both diseases.

Three molecules, namely PTN (AUC = 0.8833), CD27 (AUC = 0.875), and ANGPT1 (AUC = 0.8583), were found to potentially serve as biomarkers for PNBM with a high diagnostic efficacy. ANGPT1 was validated to have a neural protective character in stroke because of its angiogenic and antiinflammation properties (27). Another study found that ANGPT1 could be a diagnostic biomarker for ischemic stroke (IS) (28). However, except for a transcriptomic study concerning its diagnostic role in Lyme neuroborreliosis, studies on ANGPT1 with regard to CNS bacterial infection are still lacking. Similarly, PTN was also only investigated in IS and reperfusion injury and was upregulated in microglia at disease onset, providing a neuroprotective factor.

Furthermore, the membrane receptor CD27 involves adaptive immunity via regulating T and B cells (29, 30). Although CD27^+^ T cells have proven to be involved in the pathogenesis of meningitis (31), there is still a lack of direct evidence that the soluble CD27 protein is associated with PNBM or stroke. Although some molecules have been previously reported in stroke or CNS bacterial infection, others, such as CXCL-13, TNFRSF4, TNFRSF9, TNFSF14, GZMA, GZMB, GZMH, PDCD1, CXCL-11, CAIX, PTN, and EGF, have never been shown to be associated with the pathogenesis of both diseases. These molecules may offer a novel immunity profile in the pathogenesis of the comorbidity of PNBM and aSAH. We also used bioinformatics annotation for those molecules with differential expressions between the two groups. Because of the immunity-related characteristics of the proteomics panel, the majority of results of the GO analysis correlated to adaptive immunity, which was in accord with the pathophysiologic processes of PNBM.

Additionally, the pathway enrichment analysis indicated numerous signaling pathways concerning bacterial infection in the CNS, such as the TCR signaling pathway, JAK-STAT signaling pathway, IL-17 signaling pathway, and HIF-1 signaling pathway. This information provided helpful information on potential therapeutic targets in PNBM. However, the detailed mechanisms of these molecules in pathogen-induced immunity still need further investigation. Meanwhile, the diagnostic efficacy should also be validated in an augmented population.

This study has some limitations. First, because of the low positive rate of bacterial culture, the microbial types were indeed unknown. Therefore, the molecules are associated with general bacterial infections rather than with a specific type. Furthermore, because there are potential medical risks associated with CSF collection from healthy individuals, we did not obtain these samples from the control group. Therefore, results concerning the diagnostic biomarkers for PNBM are limited to patients with aSAH. Whether these molecules can be used as general biomarkers in patients with other primary neurological diseases remains to be further studied.

In summary, we identified a cohort of immunity-related molecules that can serve as potential diagnostic biomarkers for PNBM in patients with aSAH. These molecules also provide insights into the immunological profile underlying the pathogenesis of CNS bacterial infection after neurosurgical processes, thereby providing potential treatment targets for clinical practice and fundamental scientific research in this field.

## Data availability statement

The original contributions presented in the study are included in the article/Supplementary material, further inquiries can be directed to the corresponding authors.

## Ethics statement

This study involving human participants was conducted according to the Declaration of Helsinki and was approved by the Institutional Ethics Board of the First Affiliated Hospital of Anhui Medical University (Approval code: 20190146, March 2019). The patients/participants provided their written informed consent to participate in this study.

## Author contributions

LZ: study design, data collection, data analysis, and manuscript drafting. PL: study design, data analysis, and manuscript drafting. ZX: study design and data analysis. LG: methodology. XJ: data collection and data analysis. JL: data analysis and methodology. XW: supervision and sample collection. HC: study design, critical review of the manuscript, and supervision. LY: study design, data analysis, funding acquisition, and critical manuscript review. All authors contributed to the article and approved its final version for publication.

## Supplementary material

Supplementary Figure 1 Correlation coefficient analysis of the 92 proteins.
