# Supplementary figures and images for: Diagnosis of post-neurosurgical bacterial meningitis in patients with aneurysmal subarachnoid hemorrhage based on the immunity-related proteomics signature of the cerebrospinal fluid
Source: Front Neurol. 2023 Jun 20;14:1166598. doi: 10.3389/fneur.2023.1166598 (PMC10319054; doi:10.3389/fneur.2023.1166598)

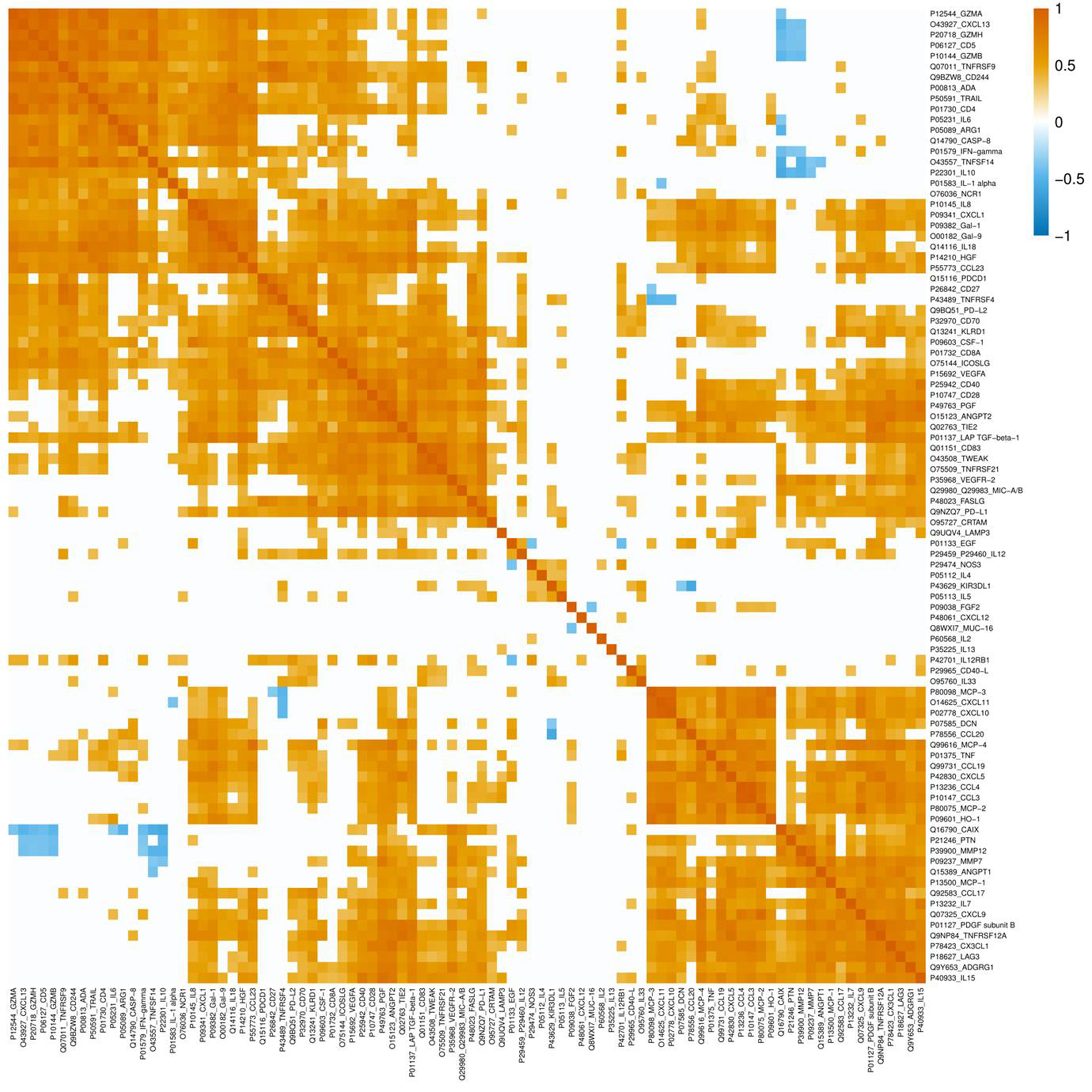

Supplement: Supplementary Figure 1 — Correlation coefficient analysis of the 92 proteins. [file Image_1.jpeg]
